# The Household Costs of Visceral Leishmaniasis Care in South-eastern Nepal

**DOI:** 10.1371/journal.pntd.0002062

**Published:** 2013-02-28

**Authors:** Surendra Uranw, Filip Meheus, Rob Baltussen, Suman Rijal, Marleen Boelaert

**Affiliations:** 1 Department of Internal Medicine, B.P. Koirala Institute of Health Sciences, Ghopa, Dharan, Nepal; 2 Department of Public Health, Institute of Tropical Medicine, Antwerp, Belgium; 3 Department of Primary and Community Care, Radboud University Nijmegen Medical Centre, Nijmegen, The Netherlands; Makerere University, Uganda

## Abstract

**Background and objectives:**

Visceral leishmaniasis (VL) is an important public health problem in south-eastern Nepal affecting very poor rural communities. Since 2005, Nepal is involved in a regional initiative to eliminate VL. This study assessed the economic impact of VL on households and examined whether the intensified VL control efforts induced by the government resulted in a decrease in household costs.

**Methods:**

Between August and September 2010, a household survey was conducted among 168 patients that had been treated for VL within 12 months prior to the survey in five districts in south-eastern Nepal. We collected data on health-seeking behaviour, direct and indirect costs and coping strategies.

**Results:**

The median total cost of one episode of VL was US$ 165 or 11% of annual household income. The median delay between the onset of symptoms and presentation to a qualified provider was 25 days. Once the patient presented to a qualified provider, the delay to correct diagnosis was minimal (median 3 days). Direct and indirect costs (income losses) represented 47% and 53% of total costs respectively. Households used multiple strategies to cope with the cost of illness, mainly mobilizing cash/savings (71%) or taking a loan (56%).

**Conclusions:**

The provision of free VL diagnosis and drugs by the Nepalese control programme has been an important policy measure to reduce the cost of VL to households. But despite the free VL drugs, the economic burden is still important for households. More effort should be put into reducing indirect costs, in particular the length of treatment, and preventing the transmission of VL through vector control.

## Author Summary

Visceral leishmaniasis (VL) is a parasitic disease and is fatal if untreated. VL mainly affects poor populations in rural areas and has important socio-economic consequences to the household because of high expenditures to access treatment and coping strategies to cover the costs of care. Since 2005, the government of Nepal has been engaged in a regional collaborative effort to eliminate VL from the Indian subcontinent. In this study we examined the economic burden of VL from the perspective of the household after the intensified implementation of VL control activities. Our findings indicate that the economic burden of VL as a percent of household income has decreased compared to studies conducted prior to the implementation of the VL elimination initiative in Nepal. In particular, the free provision of diagnosis and drugs at public health facilities has been an important policy measure. However, the economic impact of VL is still considerable and efforts are needed to further reduce the burden of VL to affected households or prevent the transmission of VL.

## Introduction

Since 2005, the Government of Nepal has been involved in a Visceral Leishmaniasis (VL) elimination programme, alongside the governments of India and Bangladesh, to reduce annual VL incidence to less than one case per 10,000 population by 2015 [1]. VL, also known as kala-azar, is a parasitic disease that is fatal if treatment is not provided timely. The disease is transmitted from humans-to-humans through the bite of a female sandfly.

Geographically, VL occurs in the alluvial plains of the river Ganges, in districts bordering the frontiers between Bangladesh, India and Nepal. The cases in this region account for 60% of the global burden of VL. In Nepal, 8 million people are at risk of acquiring VL in 12 districts in the central and eastern regions of the country [2]. Between 2000 and 2010, 17,462 cases and 244 deaths were reported in Nepal although these figures, obtained through passive case surveillance at government health facilities, are likely to be underestimations since many cases are not reported or remain undetected [3]–[5].

The cornerstones of the VL elimination initiative are early detection and appropriate treatment, in an attempt to curtail transmission of the disease. A standardized clinical case definition was adopted, the rK39 rapid diagnostic test was introduced to enable faster detection of suspected cases and miltefosine, the first oral drug for VL, replaced sodium stibogluconate (SSG) to which growing failure rates had been reported in Nepal [6]. In addition, both VL diagnosis and drugs are now provided free of charge at public health care facilities. These measures along with enhanced vector control resulted in a steady decrease in the annual number of reported VL cases in Nepal from 2,229 in 2003 to 900 in 2010. Another important outcome of the improved case management strategy may be its effect on the household costs of seeking and obtaining appropriate VL care. VL is a disease of poverty affecting the poorest of the poor [7], [8]. Households with low incomes and living in precarious housing conditions such as mud -or grass covered houses are most at risk of acquiring VL disease [[9], Uranw *et al.* unpublished data]. The few studies in Nepal quantifying the economic burden of VL on households and conducted prior to the elimination initiative showed a VL episode to profoundly impact the socio-economic status of the household. Adhikari *et al.* (2009) reported that up to 26% of previously non-poor households were pushed into poverty as a direct result of out-of-pocket expenditures on VL care while Rijal *et al.* 2006 showed that the (median) direct and indirect costs of a VL episode were equal to one year of median per capita income. Usually an expenditure exceeding 10% of annual household income is defined as catastrophic, meaning it drives households into destitution [10], [11]. These high costs are caused, amongst others, by long delays, up to 2 months, before correct diagnosis whereby households consulted traditional and private-for-profit providers [12] as well as the long hospitalization due to the use of SSG. Faster screening of suspected cases, enhanced access to free treatment, and a different treatment regimen may reduce these household costs. We examined whether the intensified VL control efforts induced by the elimination initiative resulted in a demonstrable impact at household level in terms of health seeking behaviour, costs and coping strategies.

## Methods

Nepal is administratively divided into 14 zones and 75 districts. In 2010, VL was reported in 12 districts situated in south-eastern Nepal in the Terai region bordering the highly VL-endemic northern state of Bihar in India. The study was conducted between August and September 2010 in five of these highly endemic districts, namely Siraha, Saptari, Sunsari, Morang and Jhapa. VL incidence rates in the study districts varied from 0.52 cases per 10,000 persons per year (Sunsari district) to 2.03 cases per 10,000 persons per year (Saptari district) in 2010 (table 1).

**Table 1:** Characteristics of districts included in the study.

	Siraha	Saptari	Sunsari	Morang	Jhapa
Location	Central Terai	East Terai	East Terai	East Terai	East Terai
Population, 2006	638,375	633,965	710,842	941,614	755,494
VL cases, 2006*	142	255	117	113	52
Case detection rate#	22.2	40.2	16.5	12	6.9
Cases in survey (% of notified cases in 2006)	8 (5.6)	54 (21.2)	12 (10.3)	78 (69.0)	16 (30.8)

Source: National Population & Housing Census 2011; adapted from [23].

* Notified to Epidemiology and Diseases Control Division, Nepal.

# Number of new cases reported per 100 000 person-years.

We searched the medical records of the District Public Health Office in each district and the database of the B.P. Koirala Institute of Health Sciences (BPKIHS) to identify all households in the five districts with a household member treated for kala-azar within 12 months prior to the survey. BPKIHS is a tertiary level hospital situated in Sunsari district and draws many patients from the surrounding areas due to its widespread reputation as a VL treatment and research centre. Patients treated at BPKIHS are not included in the medical records of the District Public Health Office. Furthermore, to minimize recall bias, we only considered the most recent case of VL in the household.

### Organization of VL care services

Health care services at the district level are provided by sub-health posts, health posts, primary health care centres and district hospitals (i.e. primary care level) [13]. A network of female community health volunteers at the village level refer patients to health -and sub-health posts. Patients suspected of VL (defined as individuals with a history of fever of more than 2 weeks with a palpable spleen) seeking care from female community health volunteers, sub-health posts and health posts are referred to primary health care centres or district hospitals for diagnosis by a rapid diagnostic test (rK39 immunochromatographic strip test). If positive, the patient is treated at the PHC or referred to a district hospital (or higher level) if the PHC does not have a medical doctor, which is often observed. All VL drugs are provided free of costs. Diagnosis through parasitology (bone marrow or splenic aspiration) can only be done at district hospitals or above. While private formal providers such as private clinics also provide diagnosis and treatment of VL, free treatment is only available at public facilities. Various anti-leishmanial drugs are available in Nepal: since 2006 SSG, administrated intramuscularly for 30 days, was replaced as first line treatment by miltefosine, an oral drug given for 28 days. Due to its possible teratogenic effect, miltefosine is not given to pregnant women. The second line treatment is amphotericin B deoxycholate given every day for 14 doses.

### Data collection and analysis

Information for the study was obtained from patient medical records and a household questionnaire. Medical records at the District Public Health Offices and the BPKIHS were consulted to retrieve data on the type of VL drugs received and the length of treatment. Subsequently households were visited at their homestead by a team of trained field workers who had previously been involved in other kala-azar related community and household surveys in the area. The field workers were supervised on a daily basis by the first author (S. Uranw). They used a pre-tested structured questionnaire administered to the head of the household or the most knowledgeable person. The survey collected data on treatment seeking behaviour (health providers visited, mode of travel, delay to presentation to first qualified health professional, etc.), direct and indirect costs and the coping strategies to meet the health seeking and treatment costs.

Direct medical and non-medical cost data were gathered for each provider visited. Direct medical costs included all out-of-pocket expenditures by the household on consultation, medicines and laboratory tests. Direct non-medical costs included expenditure on transportation to and from the health facility, food costs and other daily expenditures for the patient and accompanying family members. The indirect cost of a VL episode represented the loss of productivity within the household due to illness and was estimated using the human capital approach. The loss of productivity was valued in terms of the loss of earnings of the patient and household members caring for the patient (either at home or hospital). For patients and attendants, the daily wage rate was estimated and multiplied by the number of work days lost to obtain the indirect cost of a VL episode. The daily wage rate was determined by asking a series of questions on the daily monetary income (the main source of income to most household members was daily labour). For patients and attendants reporting farming as their main source of income, the survey collected data on the yearly production of each produce which was then valued with local market prices and divided by the number of agriculturally active household members. We also estimated total household income as the sum of monthly cash income from daily labour for each economically active household member, the income from agriculture, income from sales of animals and animal products (e.g. milk) and remittances from family members.

### Analysis

The data is described using descriptive statistics showing proportions, means and standard deviations. We also presented medians and interquartile ranges (25^th^ and 75^th^ percentile) because of skewed distributions in the cost data; many households reported zero out-of-pocket expenditure for some cost categories and providers, in particular informal health care providers. Costs were defined as catastrophic if they exceeded 10% of annual household income [10], [11]. All costs were converted from Nepalese rupees (Rs.) to US dollars using the exchange rate prevailing at the time of the study (1 USD = Rs. 74.8; OANDA August 2010). Data entry and cleaning were done in Microsoft Excel and analysis in STATA v10.1 (Stata Corp., College Station Tx, USA).

### Ethical considerations

Ethical clearance was obtained from the ethics committee of the BP. Koirala Institute of Health Sciences, Nepal and the ethics committee of the University of Antwerp, Belgium. Patient's medical records were reviewed retrospectively and all information retrieved from medical records was anonymized. Signed informed consent was obtained from all adult patients and from a parent or guardian of participating minors. All households that were approached for the study, whether they accepted to participate or not, received a free long-lasting insecticide treated net (Vestergaard Frandsen A/S, Denmark) as a compensation for their time spent with the survey team.

## Results

### Characteristics of study participants and the household

We randomly retrieved a total of 200 households where a case of VL occurred in the past 12 months in the five districts, of which 168 households were located by field workers and accepted to be interviewed. The majority of patients were male (60%) and 41 of them were head of the household (24%). Most patients were over 14 years of age (68%); the median age was 19 (IQR 12.5-35). Male patients were significantly older than female (median age 22.5 versus 17.0; *p*<0.05). The percentage of women of childbearing age (15–49 years) among patients was 19%. The median household size was 5.7 persons.

Out of 168 patients, sixty-one (36%) were economically active at the time of illness, most of them day labourers (80%) such as rickshaw driver or farm labourers. Few patients were engaged in small-scale farming (n = 5; 8%) or were salaried workers (n = 4; 6%). The median monthly income of an economically active patient (n = 60) was Rs. 6,000 or 81 US$ (range 27–162 US$). The median monthly income of the household (n = 168) was Rs. 10,243 or 138 US$ (range 87–163 US$) giving a median per capita monthly income of Rs. 1,882 or 25 US$ (range 20–30 US$).

The vast majority of households lived in non-permanent housing structures either consisting entirely of natural materials (49%) or a combination, usually mud walls and metal sheets as roof (45%). Eighteen per cent of households owned land (n = 30); 27 of these households cultivated some crops on their land, mainly paddy rice and wheat. Most households also owned some livestock (83%), usually goats (56% of households; median 3 heads), cows (44%, median 2.5) or chickens (36%, median 5). Fifty-eight per cent of households owned a bicycle, 56% a mobile phone and 36% a radio.

### Health-seeking behaviour

Patients visited a median of 2 health providers including the one who eventually treated them (IQR 1–2) (table 2). For 91 households (55%), a public provider was the patient's first point of contact; other households first visited a private (qualified) provider (n = 34; 20%), a traditional healer (n = 26; 15%) or a chemist or pharmacy (n = 16; 10%). The main reasons behind the choice of the first provider were proximity (49%) and the perceived (good) reputation of the health provider (38%). Traditional healers were chosen for their proximity, public providers (health centre or hospital) most often for their reputation while for private providers it was a mix of both. Ninety patients (54%) were submitted to a VL diagnostic test on their first visit but this varied considerably by type of provider: all patients presenting at public hospitals were tested for VL, compared to 56% at public health centres and 39% at private providers. Of the patients that did not receive a VL diagnostic test at a public health centre or a private provider on their first visit, respectively 60% and 55% were subsequently referred by the provider to a public hospital for testing and treatment. Households that used the services of an unqualified provider first, were more likely to visit either another unqualified provider or a private provider afterwards. Approximately 21% (n = 35) of households visited three different providers; 4% (n = 7) of households visited 4 different types of health providers.

**Table 2:** Health seeking behaviour of households (n = 168).

Variable	N° (%) of patients
**Type of health provider first visited**	
Traditional	26 (15.5)
Chemist or pharmacy	16 (9.5)
Village health worker	6 (3.6)
Public, primary	23 (13.7)
Public, hospital	63 (37.5)
Private doctor/clinic	34 (20.2)
**Delay to presentation** (in days) (median; IQR)	25 (20–30)
**Delay to diagnosis** (in days) (median; IQR)	3 (2–7)
**Number of health providers visited**	
1	63 (37.5)
2	70 (41.6)
3	28 (16.7)
4	7 (4.2)
**Diagnosed with VL at first visit?**	
Yes	90 (53.6)
No	78 (46.4)
**Mode of transportation to first facility**	
Foot	38 (22.6)
Bicycle	28 (16.7)
Bus	96 (57.1)
Other	6 (3.6)
**Mode of transportation to treatment facility** 1	
Foot	4 (2.4)
Bicycle	10 (6.0)
Motorbike	2 (1.2)
Bus	148 (88.6)
Other	3 (1.8)
**Distance between home and treatment facility (kilometers)**	
<20	45 (26.8)
20–60	86 (51.2)
>60	37 (22.0)

1 The treatment facility is the health provider where the patient received VL treatment. In 98% of cases this was a public hospital.

The median delay between the onset of symptoms and presentation to a qualified health provider (i.e. patient delay) was 25 days (IQR 20–30). Once the patient had presented to a qualified provider, the median delay to correct diagnosis of VL was 3 days (IQR 2–7). The total median delay from onset of symptoms to start of treatment was 31 days (IQR 23–35). While none of the delays varied with age or gender, there was a significant and positive relationship between the total delay and the number of providers visited (p<0. 01).

### Treatment regimens

The vast majority of patients in our study were treated with either miltefosine (83%) or conventional amphotericin B (15%) in case of relapse as recommended by the 2005 guidelines of the VL elimination initiative [1]. Four patients were treated with SSG (2%), two of them by a public provider. The other two patients treated with SSG by a private provider were unexpected in our survey because these patients are usually not included in the DPHO records. In addition, both patients reported not to have paid for the SSG drugs. Upon closer inspection, these patients were from the same village in Morang district on the border with Bihar state (India). After discussion with the local vector control officer, they had probably obtained the SSG free of charge from a private charitable hospital in Bihar and subsequently received the injections at a health facility in Nepal by trained health workers.

### Direct costs

The average and median direct household costs incurred by type of provider are given in table 3. All but three patients received VL treatment at a public hospital, the remaining 3 patients were treated at a public health centre (n = 1) or a private qualified provider (n = 2).

**Table 3:** Direct medical and non-medical costs of treatment per patient by type of provider (Rs. 2010).

	Traditional (n = 28)				Chemist/pharmacy (n = 25)				Village health worker (n = 6)			
	Mean	(sd)	Median	(IQR 25–75)	Mean	(sd)	Median	(IQR 25–75)	Mean	(sd)	Median	(IQR 25–75)
Direct medical costs												
Consultation	210	(363)	50	(0–200)	74	(76)	50	(10–100)	72	(114)	30	(10–60)
Ancillary drugs	425	(847)	0	(0–750)	1,242	(1,526)	700	(500–1,400)	657	(242)	670	(500–800)
Laboratory investigations	45	(137)	0	(0–0)	225	(218)	100	(0–400)	67	(103)	0	(0–200)
Total direct *medical* costs	679	(973)	300	(0–1,000)	2,034	(2,950)	1,150	(500–2,000)	795	(325)	785	(650–1,000)
Direct non–medical costs												
Transportation	7	(30)	0	(0–0)	113	(194)	0	(0–100)	0	(0)	0	(0–0)
Food	314	(567)	0	(0–350)	107	(149)	0	(0–200)	17	(26)	0	(0–50)
Other	0	(0)	0	(0–0)	2	(10)	0	(0–0)	8	(20)	0	(0–0)
Total direct *non-medical* costs	321	(564)	0	(0–350)	222	(319)	100	(0–200)	25	(42)	0	(0–50)
Total direct costs	1,001	(1,150)	775	(75–1,700)	2,256	(3,158)	1,150	(500–2,200)	820	(350)	785	(700–1,000)

	Public, primary (n = 32)				Public, hospital (n = 165)				Private doctor/clinic (n = 52)				Total costs all providers			
	Mean	(sd)	Median	(IQR 25–75)	Mean	(sd)	Median	(IQR 25–75)	Mean	(sd)	Median	(IQR 25–75)	Mean	(sd)	Median	(IQR 25–75)
Direct medical costs																
Consultation	56	(85)	25	(10–50)	79	(191)	50	(25–70)	222	(155)	200	(200–250)	203	(297)	88	(40–270)
Ancillary drugs	1,002	(845)	600	(500–1,345)	1,120	(1,580)	650	(400–1,080)	1,714	(1,567)	1,200	(825–2,000)	2,134	(2,137)	1,500	(700–2,850)
Laboratory investigations	317	(349)	200	(95–425)	442	(603)	300	(200–500)	748	(767)	500	(300–875)	786	(872)	600	(300–1,000)
Total direct *medical* costs	1,375	(1,107)	975	(608–1,900)	1,550	(1,703)	1,000	(720–1,688)	2,684	(2,038)	2,000	(1,475–3,575)	3,123	(2,745)	2,390	(1,100–4,290)
Direct non–medical costs																
Transportation	75	(114)	23	(0–100)	431	(343)	450	(200–500)	390	(392)	300	(150–500)	574	(481)	500	(300–800)
Food	123	(144)	100	(0–200)	1,424	(968)	1,400	(700–2,000)	414	(571)	200	(100–500)	1,619	(1,018)	1,500	(1,000–2,250)
Other	29	(97)	0	(0–0)	239	(216)	200	(100–400)	68	(183)	0	(0–0)	260	(252)	200	(100–480)
Total direct *non-medical* costs	226	(290)	125	(23–275)	2,090	(1,207)	2,000	(1,300–2,700)	882	(1,007)	600	(300–1,100)	2,453	(1,400)	2,300	(1,550–3,350)
Total direct costs	1,601	(1,266)	1,075	(693–2,260)	3,640	(2,531)	3,130	(2,135–4,223)	3,582	(2,545)	2,500	(2,000–5,000)	5,576	(3,552)	4,905	(3,025–7,125)

The median direct cost of an episode of VL across all providers was Rs. 4,905 (IQR 3,025–7,125) or US$ 66 (IQR 41–96). Direct medical costs were Rs. 2,390 (IQR 1,100–4,290) and non-medical costs Rs. 2,300 (IQR 1,550–3,350) or 51% and 49% respectively of total median direct costs. Direct medical costs arose from expenditures on consultation fees, miscellaneous drugs and laboratory investigations (including diagnostic tests). The survey confirmed that none of the households had to pay for VL drugs. Median direct medical costs were highest for households visiting private providers (median Rs. 2000; IQR 1,475–3,575), in particular payments for ancillary drugs (e.g. antibiotics, antipyretics or vitamin injections). The direct non-medical costs, consisting of transportation, food and other expenses (i.e. small daily expenses) were highest at the public hospital. The high food costs at public hospitals (median: Rs. 1,400; IQR 700–2,000) arose from the hospitalization of the patient and accompanying family member(s) for VL treatment. The median duration of hospitalisation was 10 days (IQR 7–16) and was the same for patients receiving SSG or miltefosine but higher for patients treated with conventional amphotericin B (median: 14 days; IQR 8–20). Direct (medical and non-medical) costs did not vary by gender or income quintiles, but direct medical costs increased with the patient's age.

### Indirect costs

VL is a syndrome characterized by prolonged fever, weight loss, anaemia, fatigue and enlargement of the liver and spleen. As a result patients are either severely limited or not able at all to carry out their daily activities and need much support from family members. Among the 168 patients, 95% (n = 160) reported that VL illness had a severe impact on their normal functioning and resulted in a loss of income to the household, either wage losses to the patient or caretakers, losses in agricultural output or other earnings. Patients reported not being able to carry out their normal daily activities for a median number of 57 days (IQR 51–65) (table 4). As a result the median loss of income was Rs. 12,400 for economically active patients. Since only 36% of patients were economically active, the value of time lost across all patients, both the economically active and non-active, was on average Rs. 4,731.

**Table 4:** Indirect costs (Rs. 2010).

	Mean (sd)		Median (IQR 25–75)	
Patients' duration of illness (days)§	60	(18)	57	(51–65)
Number of attendants per patient	1.1	(0.3)	1.0	(1.0–1.0)
Workdays lost by attendants	21	(16)	15	(10–30)
Loss of income; working patients only (n = 61)	13,030	(5,638)	12,400	(9,800–15,400)
Loss of income; all patients (n = 168)	4,731	(7,136)	0	(0–10,700)
Loss of income working attendants only (n = 134)	3,112	(2,300)	2,583	(1,500–4,000)
Loss of income; all attendants (n = 183)	2,279	(2,404)	1,500	(0–3,100)
*Total loss of income to the household* £	*7,213*	*(7,217)*	*4,500*	*(1,500–12,167)*
Total payment on loan*	2,611	(2,176)	2,080	(1000–3,300)
Total indirect cost	8,084	(7,391)	5,167	(3,000–13,290)

§ Consists of the various types of delays plus the treatment duration.

£ Across all patients & attendants.

* For those with interest payments.

Patients were attended by on average 1.1 household members (range: 1–2). These caretakers reported a median loss of 15 workdays (IQR 10–30) mainly due to accompanying the patient to the various health providers and staying with him/her for the full duration of hospitalization. The median loss of income to caretakers was Rs. 2,583 (on average Rs. 2,279 across all caretakers). The median total value of time lost to the household per episode of VL was Rs. 4,500 (IQR 1,500–12,167).

### Coping strategies

Households used a number of strategies to cope with the costs of VL illness. Many of these strategies resulted in additional costs to the household; e.g. in terms of interest payments on loans or hiring labour to replace the sick household member. The survey identified three strategies to cope with the financial costs of VL illness: mobilizing cash/savings, taking a loan and sales of livestock. Mobilizing cash or savings was the most frequent coping strategy. Seventy-one per cent (n = 120) of households used their savings to pay for health care expenditures, although for 54% of these households it was not enough to cover all medical costs. Out of those 120 households, 75% (n = 90) of households reported that the savings were supposed to buy food, in other cases assets (n = 30). Fifty-six per cent (n = 94) of households took a loan to finance the costs of care, most often from a member of the same village (71%), followed by friends or peers (17%) or an informal money lender (6%). When borrowing from friends, the loan was interest free. In other cases (n = 57), the amount to be repaid was on average 140% the original amount borrowed, usually through monthly instalments. A collateral was not often provided for the loan: four households provided assets as collateral, one household that took a loan from a bank provided their house. Seventeen per cent of households sold livestock to cover the costs of care (n = 29). Forty-two per cent of households chose more than one strategy (n = 71), mainly using cash/savings and a loan (n = 47), while 2% (n = 4) had to revert to all three strategies to cope with the costs of VL illness.

Households also used various strategies to compensate the labour lost due to VL illness. Nine households hired external labour to replace either the patient or caretakers in the field at a rate of Rs. 200 per day for a median of 60 days (IQR 60–60). Twenty-three per cent of patients were replaced by a family member that was a school-going child for the duration of their illness (n = 14).

## Discussion

During the past five years, considerable efforts were made by public health authorities in Nepal towards the elimination of VL such as the decentralisation and provision of free diagnosis and treatment and the introduction of the oral drug miltefosine in the public health system. In this study we studied the health seeking behaviour and documented household costs and coping strategies for one episode of VL from the perspective of the patient in a miltefosine-based treatment program. From our findings, the following observations can be made.

First, our results showed that the cost of a VL episode to patients and their family was high notwithstanding the free provision of drugs and diagnostics by the government. With a median total cost of US$ 165 per episode, the economic burden of VL across all households was 11% of annual household income or 57% of median annual per capita income (table 5). This cost included both direct costs (medical and non-medical out-of-pocket expenditures) and indirect costs (productive time losses due to illness). While about half (51%) of the households exceeded the catastrophic threshold of 10% of annual household income, it would be wrong to conclude that the economic consequences of VL illness were not significant for the other households. Because VL is a disease of poverty primarily affecting the poorest income groups [8], the ability to cope with the costs of VL illness are limited. This was evident from the coping strategies households used whereby a majority of households were forced to take a loan to pay for the costs of care and/or use all their savings. However, without the free provision of VL drugs the median cost of an episode of VL would be US$ 226 and the proportion of households exceeding the catastrophic threshold would increase from 51% to 74% (assuming a drug cost of US$63 for a 28-days course of miltefosine at WHO preferential prices [14]).

**Table 5:** Summary of direct and indirect costs per VL episode (Rs. and in US$ 2010).

	Mean		Median	
Item	Rs.	US$	Rs.	US$
Direct and indirect costs:				
- Direct medical cost	3,116	42.2	2,385	32.3
- Direct non-medical costs	2,444	33.1	2,297	31.1
- Indirect cost	8,084	110.7	5,167	70.8
*Total household costs*	13,659	187.1	12,050	165.1
Annual income:				
- Household	127,074	1,720.7	122,665	1,661.0
- Per capita	23,366	316.4	22,539	305.2
Median costs as a % of:				
- Annual household income	11%			
- Annual per capita income	57%			

Exchange rate 1 US$ = 74 Rs. (Sept. 2010).

Secondly, direct costs accounted for 47% of total costs and were largely caused by out-of-pocket expenditures households made on ancillary drugs and food. Households that visited private-for-profit health providers incurred substantial expenditures on ancillary drugs. These ancillary drugs, most frequently antibiotics, antipyretics or vitamin injections, were given to patients prior to their referral to a public hospital for VL treatment. From our study we cannot say whether the prescription of these ancillary drugs were justified on medical grounds. Besides the ancillary drugs, another important direct cost component were the high food costs for the patient and accompanying relatives and was caused by the extensive stay at the hospital for treatment. Although miltefosine is an oral drug, patients stayed at the hospital for a median of 10 days.

A number of studies had been carried out prior to the VL elimination initiative [12], [15], [16]. Our findings seem to suggest that, compared to these studies, the economic burden of VL (as a % of household income) has decreased. In particular the magnitude of indirect costs (as a % of total costs) was less in our study likely due to a shorter patient delay. We cannot say whether the shorter patient delay observed in this study was the result of increased patient knowledge of VL, but the previous studies reported that traditional providers were most commonly the patient's first choice of provider, which may have lengthened the delay until VL diagnosis. However, these conclusions need to be considered as tentative. For instance the total cost of VL in our study was higher compared to Rijal *et al*. (2006) but much lower compared to Adhikari & Maskay (2005) and Adhikari *et al.* (2009) while the household income of VL affected households in our study was higher compared to all three studies (to enable comparisons the cost data in these articles were adjusted to the year 2010 using the consumer price index [17]). While daily wages in Nepal have increased over the years, partly to compensate for the high inflation rate, methodological differences between the studies and in particular the small sample sizes of the first two studies (respectively 18 and 7 households) limit their generalizability beyond the communities or villages where these studies were carried out.

Despite the provision of free VL drugs, we have shown that households still incurred substantial medical out-of-pocket expenditures, especially at private providers. It remains to be seen, however, if these medical costs can be prevented or at least diminished. While prepayment schemes, such as community-based health insurance, may be a solution, coverage in Nepal is very low and expansion of coverage to VL affected households is unlikely in the near future. More realistic and feasible approaches consist of reducing indirect costs and vector control. Treatment duration can be reduced substantially by considering alternative VL drugs to miltefosine as single-dose liposomal amphotericin B or a short course combination therapy [18]). Vector control, such as indoor residual spraying, has been shown to be effective in reducing the number of sandflies inside the house [19], [20] and may therefore reduce disease transmission. Since 2011, the Government of Nepal has also introduced a conditional cash transfer programme whereby households receive Rs. 1,000 (US$13.5) upon completion of treatment at a public hospital [21]. This payment can be used to cover transportation costs but was not yet in place when we carried out the household survey. However from our study, the total transportation costs of households were smaller than Rs. 1,000 while food costs were higher. The conditional cash programme therefore ought to be expanded to include food costs as well.

This study had a number of limitations. Because patients were selected from the medical records of the District Public Health Offices and the BPKIHS, only patients treated at public health facilities were included in the study. The records kept by the District Public Health Offices are obtained through passive surveillance from cases detected and treated by public health facilities. Private for-profit providers are not required to report patients treated at their facilities. Due to the low incidence of VL and the high number of private practioners in Nepal, it would have been difficult and costly to find and interview these patients. Because patients exclusively treated at private-for-profit providers were excluded, we probably underestimated the true burden of VL. Despite this limitation, our findings were still representative for a large proportion of the VL population because in Nepal, contrary to India, a relatively small proportion of patients seek VL treatment from private-for-profit providers (about 11% of patients according to [22]. Nonetheless, efforts should be made in the future to include the private sector in the control of VL.

A second limitation is related to the recall bias. With decreasing VL incidence rates in Nepal, we have chosen a recall of 12 months to allow the identification of a sufficient number of households. To minimize the recall bias we only analysed the most recent case of VL in the household. Because of the clustering of VL in communities and villages, several cases of VL often occur in the same household. For instance, in 44% of households in our study one or more members had been treated for VL before, often within a few years. The occurrence of more than one case of VL in the same household would significantly increase the economic impact of VL to households. And even if these cases do not occur in the same year, the risk of impoverishment and indebtedness would still be much higher.

### Conclusion

With a shorter delay to diagnosis once the patient has presented to a qualified health provider and the free provision of the correct first-line drug, our study indicates that the case management component of the Nepalese VL programme performs rather well. In particular free VL diagnosis and drugs at public health facilities have been an important policy measure in Nepal to lower financial barriers and improve access to VL diagnosis and care. Without this policy the economic burden of VL would have been much higher. However, the economic impact of VL is still considerable and intensified efforts are needed to further reduce the burden of VL to affected households or prevent the transmission of VL. These include shortening the duration of stay at the hospital and expanding demand side financing mechanisms to cover a wider range of costs incurred by households.
